# Are natural estrogens used in contraception at lower risk of venous thromboembolism than synthetic ones? A systematic literature review and meta-analysis

**DOI:** 10.3389/fendo.2024.1428597

**Published:** 2024-08-16

**Authors:** Jonathan Douxfils, Lucie Raskin, Marie Didembourg, Nathalie Donis, Jean-Michel Dogné, Laure Morimont, Charlotte Beaudart

**Affiliations:** ^1^ Qualiblood sa, QUALIresearch, Namur, Belgium; ^2^ Department of Pharmacy, Clinical Pharmacology and Toxicology Research Unit, Namur Research Institute for Life Sciences (NARILIS), University of Namur, Namur, Belgium; ^3^ Department of Biological Hematology, Centre Hospitalier Universitaire Clermont-Ferrand, Hôpital Estaing, Clermont-Ferrand, France

**Keywords:** meta-analysis, combined oral contraceptive, estradiol, ethinylestradiol, venous thromboembolism

## Abstract

**Background:**

Venous thromboembolism (VTE) poses a significant global health challenge, notably exacerbated by the use of combined oral contraceptives (COCs). Evidence mainly focuses on the type of progestogen used in COCs to establish the increased risk of VTE with less data assessed on the type of estrogen used. This meta-analysis aims to assess the risk of VTE associated with COCs containing synthetic estrogens like ethinylestradiol (EE) versus natural estrogens like estradiol (E2).

**Methods:**

A systematic review and meta-analysis was conducted following the 2020 Preferred Reporting Items for Systematic Reviews and Meta-Analyses (PRISMA) guidelines. Literature searches were performed in December 2023 in MEDLINE and EMBASE to identify clinical studies comparing the VTE risk between COCs containing synthetic versus natural estrogens. Studies were selected through rigorous screening, and data extraction followed standardized protocols, with statistical analyses employing a random effects model.

**Results:**

The search yielded five relevant studies, involving over 560,000 women/time, demonstrating a significant 33% reduction in VTE risk among users of natural estrogen-based COCs compared to synthetic estrogen-based COCs (OR 0.67, 95% CI 0.51–0.87). Stratification analyses using adjusted hazard ratios (HR) of the main observationnal studies showed a 49% reduced VTE risk of E2-based pills compared to EE in association with levonorgestrel.

**Discussion and conclusion:**

Despite the longstanding use of EE-based COCs, emerging evidence supports a lower thrombotic risk associated with natural estrogens. This meta-analysis substantiates the lower VTE risk associated with natural estrogen-based COCs compared to synthetic alternatives, advocating for a re-evaluation of contraceptive guidelines to prioritize patient safety and reduce thrombotic risks.

## Introduction

Venous thromboembolism (VTE) represents a significant health concern worldwide, characterized by the formation of blood clots in the veins, which can lead to potentially fatal conditions such as deep vein thrombosis (DVT) and pulmonary embolism (PE) (1). Among the various risk factors identified for VTE, the use of combined hormonal contraceptives (CHCs) has been a subject of extensive research and debate within the medical community (2). CHCs, which typically contain a combination of an estrogen and a progestogen, are among the most effective and widely used methods for preventing pregnancy (3). However, their association with an increased risk of VTE has prompted a revaluation of their safety profile (4). The conclusion of these previous evaluations revealed that the dose of ethinylestradiol (EE) and the associated progestogen were the determinants of this increased risk of VTE (5).

Epidemiological studies have played a pivotal role in quantifying the risk of VTE associated with CHCs use (6, 7). The incidence of VTE among women of reproductive age not using CHCs is estimated to be about 2 per 10,000 woman-years (2, 4). In contrast, women using CHCs containing EE have been shown to experience a 3- to 6-fold increase in the risk of developing VTE, translating to an incidence rate of approximately 5–12 per 10,000 woman-years, depending on the type of progestogen and the dose of estrogen (2, 4). In Europe and in the United States, this may translate into additional 20,000 cases of VTE each year in this young and usually healthy population. These findings have been consistent across various studies, including those requested by regulatory agencies such as the U.S. Food and Drug Administration (FDA) (8)and the European Medicines Agency (EMA) (4, 9–13), which have issued warnings and guidelines regarding the use of CHCs and their associated risk of VTE (14).

The risk of VTE with CHCs use is further influenced by several factors, including individual risk factors such as age, smoking, obesity, and a personal or family history of VTE (14–16). While some of these individual risk factors are difficult to mitigate, the prescription of safer CHC has emerged as the preferred option among clinical practice (14). Therefore, combined oral contraceptives (COCs) using an association of EE with levonorgestrel or norgestimate has been perceived as the safer choice for first-line contraception (14). Nevertheless, recent evidence tends to confirm a previous observation that the type of estrogen is also a critical factor in the risk of VTE associated with COCs (16, 17). Indeed, EE, a synthetic estrogen, is the most used estrogen component in CHCs since the 60s and despite its doses have been lowered over the years to reduce the risk of VTE, its replacement as the estrogenic component of pills has only been proposed in the late 2000s. Consequently, most epidemiological studies, collecting data over several years, are reporting and comparing the safety of pills containing the same main driver of the VTE risk, i.e. EE (4, 6–8).

Estradiol (E2)-based COCs and the recent association of estetrol (E4) with drospirenone (DRSP) are considered to offer a more physiological approach, potentially translating to a lower risk of VTE. Preliminary studies and pharmacological data suggest that E2 and E4, two natural estrogens, may exert a less pronounced effect on coagulation factors and the hemostatic system compared to ethinylestradiol, supporting a potentially lower risk of VTE (18–22). However, the evidence comparing the VTE risk between ethinylestradiol-containing COCs and those using natural estrogens is still evolving, with some studies suggesting a reduced risk while others report no significant difference (13, 23–25). To better understand these results, it is important to note that all post authorization safety studies (PASS) uses EE-based pills comparators and therefore, although a point estimate may show a reduced VTE risk tendency, these are not statistically significant due to a lack of power inherent to the design of these studies. Given the significant health implications of VTE and the widespread use of CHCs, a thorough understanding of the risk associated with different type of estrogen is paramount.

This meta-analysis aims to critically assess the existing literature and provide a comprehensive comparison of the risk of VTE associated with synthetic estrogens versus natural estrogens. By synthesizing data from various observational studies comparing ethinylestradiol with natural estrogens, this analysis seeks to clarify the relative safety profiles of these CHCs and guide clinical practice in contraceptive choice. We aim to offer evidence-based recommendations that can inform healthcare providers and users about the safest contraceptive options in terms of VTE risk.

## Materials and methods

This systematic review was conducted in accordance with the 2020 Preferred Reporting Items for Systematic Reviews and Meta-Analyses (PRISMA) as well as PRISMA-Search for literature searches (26). In this context, a protocol for the systematic review/meta-analysis has been registered in Open Science Framework (ID https://osf.io/n9dav/). The protocol has been amended in April 2024 to include additional stratification analyses.

### Literature search

The electronic databases MEDLINE (via Ovid) and EMBASE were searched in December 2023 for any epidemiology clinical study reporting risk of VTE associated with synthetic estrogens versus natural estrogens. The search strategy employed in the two databases is available in **Appendix 1**. Additionally, a manual search within the bibliography of relevant papers was performed in order to complete the bibliographic search. Experts in the field were contacted to provide any missing references. Finally, previous systematic reviews and meta-analyses on a similar topic were also searched for backward/forward referencing.

### Study selection

The search results from the electronic sources and hand searching were imported into Covidence software for data management. Covidence is a web-based collaboration software platform that streamlines the production of systematic and other literature reviews. During the initial screening phase, three reviewers (J.D., C.B, L.R.) independently assessed the title and abstract of each obtained reference to eliminate articles irrelevant to the systematic review. Rigorous inclusion criteria were applied, as outlined in **Table 1**. In the subsequent step, the three reviewers individually examined the full text of each article that had not been excluded in the initial stage, selecting studies that fulfilled the inclusion criteria. Any discrepancies in article selection were resolved through discussion and consensus.

**Table 1:** Inclusion criteria.

PICO(S) criteria	
**Patients**	Women, 18 years and older
**Intervention**	Natural estrogens: Estradiol, Oestradiol, Estradiol Valerate, Oestradiol Valerate, Estetrol, Oestetrol
**Comparator**	Synthetic estrogens: Ethinylestradiol or Ethinyloestradiol + any progestative (e.g. levonorgestrel, norgesterel, desogestrel, gestodene, drospirenone, etc.).
**Outcome**	Risk of thrombosis Any type of venous thrombosis (suspected, confirmed, idiopathic)
**Study design**	Longitudinal observational studies: prospective or retrospective cohort studies Case-control studies

### Data extraction

Data extraction and analysis followed a clearly defined process. First, all included papers were summarized using a standardized data extraction form. This extraction form was pretested by two reviewers with three studies. Relevant insights of these studies were used to adjust the standard data extractions form. The following data were then extracted by one reviewer (J.D.) and double checked by a second reviewer (C.B.): 1) study characteristics including first author, year of publication, sample size and characteristics (age, BMI, ethnicity, comorbidity, duration of estrogens use); 2) intervention characteristics including type of estrogen (natural vs synthetic), composition, route of administration; 3) outcomes including type of thrombo-embolic events, diagnostic methods, raw prevalence data, or effect sizes (Odds Ratio (OR) or Hazard Ratio (HR) raw or adjusted). Authors were contacted in case of missing or incomplete data.

### Quality appraisal

Quality of the cohort and case-control was measured using the Newcastle Ottawa Scale (NOS) for cohort studies or nonrandomized studies.

### Statistical analyses

The risk of VTE associated with synthetic estrogens versus natural estrogens in each individual studies were pooled together using a random effect model meta-analysis. This specific model was used because heterogeneity was *a priori* expected. Peto odds ratio and their respective 95% Confidence Intervals (CI) were reported as effect size, to account for the low incidence of events, as recommended by the Cochrane Handbook for Systematic Reviews of intervention (27). When adjusted HR were provided by authors, pooled HR were computed with random effect model. In the global meta-analysis model, all E2-based COCs (E2-NOMAC, E2-dienogest (DNG)) were pooled together and compared to all EE-based COCs pooled together (EE-levornogestrel (LNG) or other COCs – (oCOC), i.e. COC containing EE and progestogens other than LNG). Subgroup analyses were further performed according to the type of EE-based COCs (E2-based COCs versus EE/LNG and E2-based COCs vs oCOC) and according to the regulatory status of the study (i.e. imposed PASS (13, 23),). Finally, a comparison of risk between EE-based COCs (EE/LNG vs oCOC) was also performed as explanatory analysis. To allow statistical comparisons between studies, all events were reported as event per person-time for prospective studies or even per number of participants for case-control studies. Only confirmed VTE were considered as events. For studies reporting the outcomes for multiple follow-up time periods, the outcomes reported for the longest follow-up time period were used in the general model. Results were examined for heterogeneity using Cochran’s Q statistic and the I² statistic. A leave-one-out sensitivity analysis was performed to evaluate the stability of the results when one study is removed at a time. Due to the limited number of studies included in the model, publication bias could not be assessed. For all results, a two-sided p value of 0.05 or less was considered as significant. All analyses were performed using R Software and appropriate packages (meta, metafor).

### Assessing the strength of evidence: GRADE

For all associations determined by meta-analyses, the level of evidence was evaluated using the GRADE (Grading of Recommendations, Assessments, Development and Evaluation) assessment. Due to the observational nature of the data, the evidence score started at moderate level of evidence and was downgraded by one (i.e. low level of evidence), two or three levels (i.e. very low level of evidence) if one of the following pre-specified criteria was present: 1) Study quality (i.e. low study quality for more than 75% of the included studies); 2) Inconsistency (i.e. unexplained substantial significant heterogeneity); 3) Indirectness (i.e. presence of factors that limit the generalizability of the results); 4) Imprecision (i.e. large 95% IC), 5) Publication bias.

## Results

**Figure 1:**
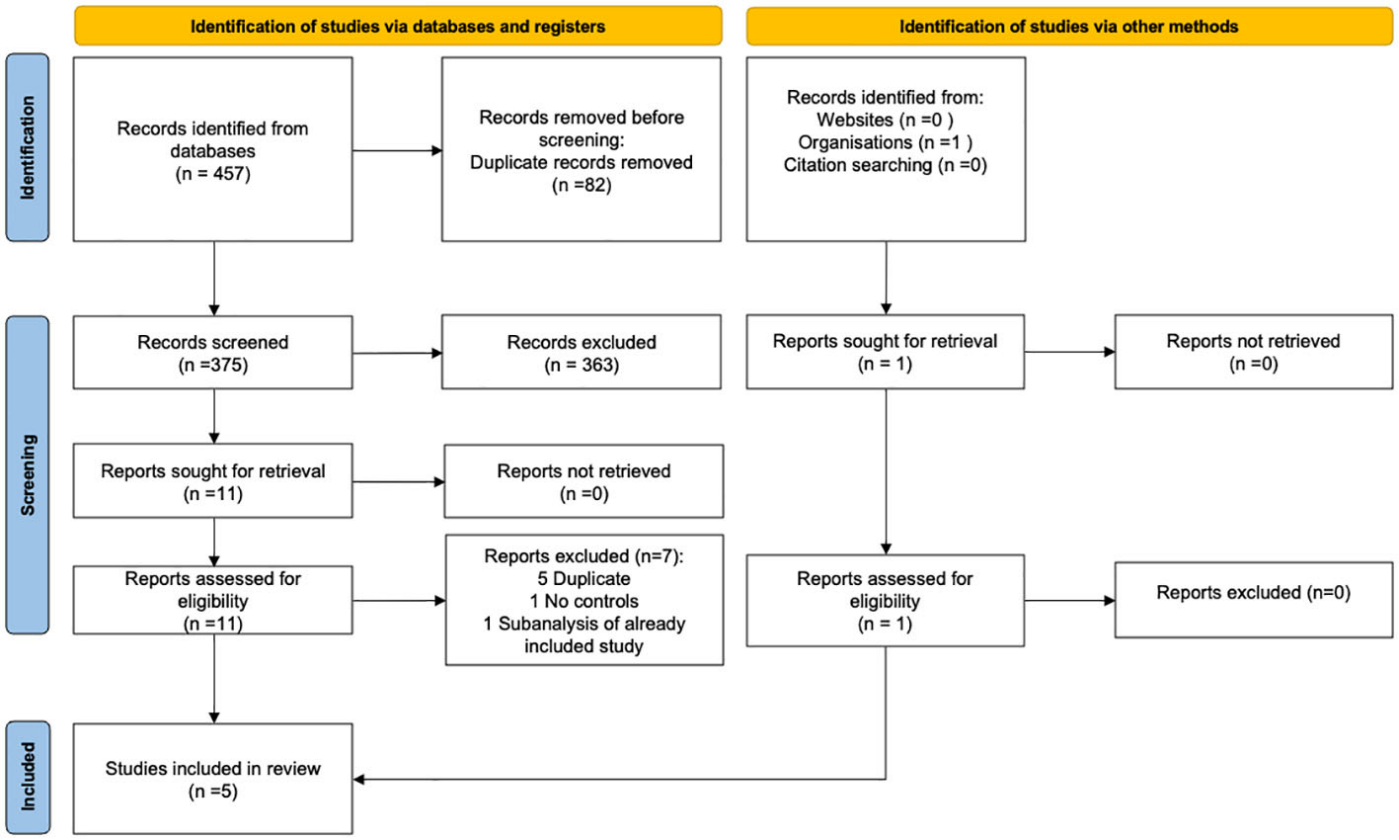
PRISMA flowchart.

The search strategy initially identified 457 records (139 from Medline and 318 from Embase), with 375 records remaining after removing duplicates. Upon screening the titles and abstracts of these records, 11 were considered potentially eligible. Following a thorough evaluation of the full texts of these articles, 7 were excluded (5 duplicates (28–32), 1 because of the lack of a control population (33) and 1 being a sub-analysis of another study already included (34) – for this study [i.e. INAS-SCORE study], E2/DNG and EE/LNG data were extracted from Bauerfeind et al. (23) while data on oCOC were extracted from Dinger et al. (34) as there were not reported in Bauerfeind et al.) Thus, a total of 4 studies met the eligibility criteria and were included in this systematic review (13, 23–25). Manual searches and expert solicitations allowed us to identify 1 additional study (35). Ultimately, a total of 5 studies were deemed relevant for inclusion in this systematic literature review. The flowchart of the study selection process is presented in **Figure 1**.

### Study characteristics

Characteristics of included studies are presented in **Table 2**. Three cohort studies and 2 case-control studies, published between 2013 and 2024, included a sample size comprised between 639 and 310,373 women. Four included studies had an excellent methodological quality with three studies rated 9/9 stars on the NOS scale and one study rated 7/9 stars on the NOS scale (13, 23–25). One study cannot be assessed due to insufficient information (abstract from congress with oral presentation) (35).

**Table 2:** Characteristics of the included studies.

Author, year	Study design	Sample size	Population characteristics: mean age, mean BMI, % smokers, % of family history of VTE	Name of COC	Event/person time – Event/number of participant^a^	Funding	Study quality (NOS scale)
Lidegaard, 2013 (35)	Prospective study	Women on E2-based COC: n=5,202 Women on synthetic estrogens: n=305,171	NR	E2-based COC: E2-DNG Synthetic estrogens: EE-LNG	E2-based COC: - 5/5,202 Synthetic estrogens: - 186/305,171	NR	Not assessed
Reed, 2021 (13)	Prospective study	E2-based COC: - n=44,559 Synthetic estrogens: - n=49,754	mean age: 30.1 mean BMI: 23.3 21% of smokers 2.4% of family history of VTE	E2-based COC: - E2-NOMAC Synthetic estrogens: - EE-LNG	E2-based COC: - 12/48,846 Synthetic estrogens: - 25/62,337	Merck Sharp and Dohme, a subsidiary of Merck & Co., Inc., Kenilworth, NJ, USA, and Theramex Ireland Limited, Dublin, Ireland	9/9 stars
Schink, 2022 (25)	Case-control study	Women on E2-based COC: - n=35 Synthetic estrogens: - n=2,512	VTE cases: mean age 17.6 20.6% obese 4.12% of smokers 10.6% of family history of VTE Controls: mean age 17.6 6.67% obese 2.54% of smokers 1.43% of family history of VTE	E2-based COC: - E2-DNG Synthetic estrogens: - EE-LNG	E2-based COC: - 6/101 Synthetic estrogens: - 1,139/12,338	Funded by the Federal Institute for Drugs and Medical Devices (Bundesinstitut f̈r Arzneimittel und Medizinprodukte, BfArM)	9/9 stars
Heikinheimo, 2022 (24)	Nested Case-control study	E2-based COC: - n=129 Synthetic estrogens: - n=510	mean age - not reported mean BMI - not reported % of smokers - not reported % of family history of VTE - not reported	E2-based COC: - E2/DNG - E2/NOMAC Synthetic estrogens: - EE/DSG - EE/GSD - EE/DRSP - EE/norelgestromin - EE/etonogestrel	E2-based COC: - 25/129 Synthetic estrogens: - 158/510	Erkko Foundation; Yrjö Jahnsson foundation (ET); Avohoidon tutkimussäätiö & Helsinki University Library	7/9 stars
Bauerfeind, 2024 (23)	Prospective study	E2-based COC: - n=11,616 Synthetic estrogens: - n=58,693	mean age 27.1 mean BMI 24.2 23% of smokers 3.1% of family history of VTE	E2-based COC: - E2-DNG Synthetic estrogens: - EE-LNG - oCOC	E2-based COC: - 11/17,932 Synthetic estrogens: - 99/107,586	Unconditional grant from Bayer AG, Germany	9/9 stars

a Event/person time reported for prospective studies – Event/number of participants reported for case-control studies.

b Only confirmed VTE were considered as events.

NR, Not Reported; COC, Combined Oral Contraceptives; BMI, Body Mass Index.

### Meta-analytical model

#### Crude analyses

**Figure 2:**
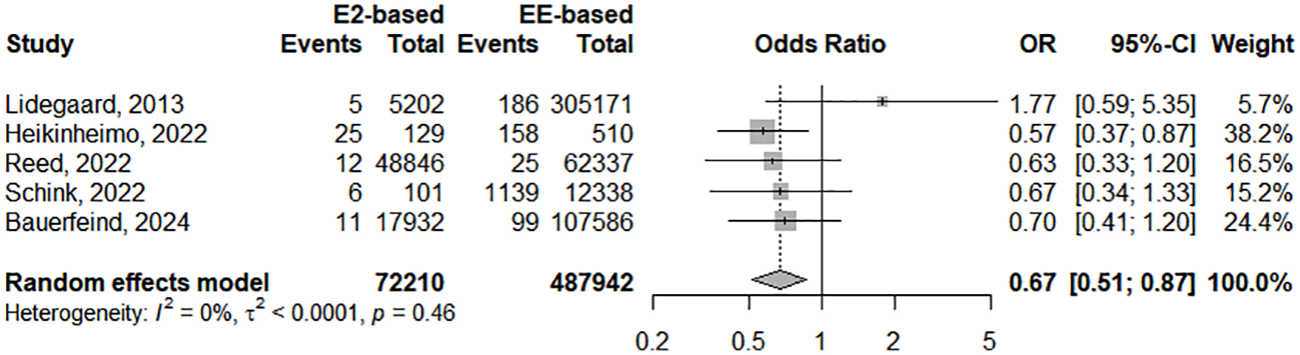
Forest plot of the studies included in this meta-analysis assessing the risk of VTE associated with natural estrogens (estradiol and estradiol valerate) versus synthetic estrogens (ethinylestradiol). CI, confidence interval; E2, estradiol; EE, ethinylestradiol; OR, odds ratio.

The global random-effect model meta-analysis, including five studies and 560,152 women/time, revealed an ORpeto of 0.67 (95%CI 0.51, 0.87), highlighting a significant 33% reduction in VTE risk among users of E2-based COCs compared to those using EE-based COCs (**Figure 2**). The model was free of heterogeneity (I²=0%, p=0.46). The leave-one-out analysis revealed an effect size ranging from 0.63 (95%CI 0.48, 0.82) when omitting Lidegaard et al. to 0.74 (95%CI 0.53, 1.03) when omitting Heikinheimo et al. The observed association was considered with a moderate level of evidence (**Table 3**). When stratifying analyses by type of EE-based COC, no significant association was found when comparing E2-based COC versus EE/LNG (ORpeto of 0.80 (95%CI 0.54, 1.17), I²=6%, p=0.36, moderate level of evidence). A significant reduction of VTE risk was found for E2-based COC versus oCOC (ORpeto of 0.60 (95% CI 0.45, 0.81), I²=0%, p=0.68, moderate level of evidence). No significant difference of VTE risk was observed when comparing EE/LNG vs oCOC (ORpeto 1.32, 95%CI 0.79, 2.21, I²=76%, p=0.02, low level of evidence).

**Table 3:** GRADE assessment per meta-analytical model.

Type of analysis	Number of studies, sample size	Random effect model (crude Peto OR, [95%CI])		Heterogeneity test (I², p for Q statistics)^b^	GRADE assessment^a^					
					Risk of bias	Inconsistency	Indirectness	Imprecision	Publication bias	Level of Evidence
E2-based COC vs EE-based COC	k=4 n E2-based= 72,210 n oCOC= 487,942	0.67 [0.51;0.87]		I² 0%, p=0.46	Not serious	Not serious	Not serious	Not serious	Not serious	Moderate
E2-based vs oCOC	k=4 n E2-based= 67,008 n oCOC= 97,082	0.60 [0.45;0.81]		I² 0%, p=0.68	Not serious	Not serious	Not serious	Not serious	Not serious	Moderate
E2-based vs EE/LNG	k=4 n E2-based= 72,081 n oCOC= 390,860	0.80 ([0.54;1.17]		I² 6%, p=0.36	Not serious	Not serious	Not serious	Not serious	Not serious	Moderate
EE/LNG vs oCOC	k=3 n EE/LNG= 85,689 n oCOC=96,572	1.32 [0.79;2.21]		I² 76%, p=0.02	Not serious	Serious	Not serious	Not serious	Not serious	Low
Additional analyses in studies providing adjusted HR										
Type of analysis	Number of studies, sample size	Random effect model (crude Peto OR, [95%CI])	Random effect model (Adjusted HR, [95%CI])^c^	Heterogeneity test (I², p for Q statistics)^b^	GRADE assessment^a^					
					Risk of bias	Inconsistency	Indirectness	Imprecision	Publication bias	Level of Evidence
E2-based vs EE/LNG in studies with adjustment for confounding factors	k=3 n E2-based= 66,811 (E2/DNG) 66,834 (E2/NOMAC) n EE/LNG= 85,689	E2/DNG 0.64 [0.41;1.00] E2/NOMAC 0.74 [0.48;1.15]	E2/DNG 0.49 [0.29;0.85] E2/NOMAC 0.68 [0.37;1.25]	E2/DNG: I²=0%, p=0.79 E2/NOMAC: I²=37%, p=0.20	Not serious	Not serious	Not serious	Not serious	Not serious	Moderate
Studies from ZEG E2-based vs EE/LNG	k=2 n EE/LNG= 66,778 n oCOC=83,177	0.64 [0.40;1.02]	0.51 [0.29;0.90)	I²=0%, p=0.66	Not serious	Not serious	Not serious	Serious	Not serious	Low

a GRADE assessment: 1) Study quality was considered as serious if low study quality was reported for more than 75% of the included studies, 2) Inconsistency was considered as serious in case of unexplained substantial significant heterogeneity, 3) Indirectness was considered serious if presence of factors that limit the generalizability of the results, 4) Imprecision was considered serious if k<3 or large 95% Confidence Intervals, 5) Publication bias: due to the low number of included studies (k=5 in the global meta-analytical model), a proper evaluation of publication bias could not be performed. However, because of the quality of the manual search performed for this systematic review and meta-analysis, any missing of evidence was considered as very unlikely and no serious publication bias was considered in the GRADE assessment.

b I^2^ is reported for adjusted HR when available, otherwise it is reported for crude Peto OR.

c Potential confounders considered in analyses: Reed et al.: age, body mass index, family history of VTE and current duration of HC use; Schink et al.: age at cohort entry, cardiovascular diseases, coagulation disorders and other blood diseases, diabetes or use of antidiabetics or insulin; migraine with aura, Varicose veins of lower extremitie, obesity, Paresis, hospitalization, surgery, fractures or trauma, Current use of ASA, antiplatelets, antithrombotics or DOACs, Current use of NSAIDs, current use of glucocorticoids or other corticoids, Current use of antidepressants or antipsychotics; Bauerfeind et al.: age, body mass index (BMI), duration of current hormonal contraceptive use and family history of VTE; for ATE, it included age, BMI, smoking, treated hypertension and a family history of fatal ATE.

#### Adjusted analyses

Adjusted HR were available for 3 studies comparing E2-based COC with EE-LNG (13, 23, 25). One study (25), reported two groups with E2-based COC (i.e. E2/NOMAC and E2/DNG) and therefore analyses where run separately to avoid inclusion of the EE-LNG arm twice in the analysis. The adjusted HR ranged from 0.49 (95%CI 0.29–0.85 I²=0%, p=0.79, with E2/DNG in the study of Schink et al.) to 0.68 (95%CI 0.37–1.25, I²=37%, p=0.20 with E2/NOMAC in the study of Schink et al.). Pooled HR from imposed EMA PASS yielded a statistically significant reduction of the HR (HR of 0.51 (95%CI 0.29–0.90), I²=0%, p=0.66). The corresponding crude OR were 0.64 (95%CI 0.41–1.00, I²=0%, p=0.98), 0.74 (95%CI 0.48–1.15, I²=26%, p=0.26) and 0.64 (95%CI 0.40–1.02, I²=0%, p=0.87). Detailed Forest plots of all analyses are available in **Appendix 2**.

## Discussion

This systematic review and meta-analysis aimed to address evolving concerns regarding the safety profile of COCs, particularly focusing on the thrombotic risk associated with their estrogen components. Despite reductions in EE dosage and the introduction of new progestins, the thrombotic risk associated with COCs remained a public health safety concern since the 60s (2). This led to the marketing of E2-containing COCs, which were presumed to have a more favorable impact on coagulation profiles due to their lesser effect on the synthesis of hepatic proteins (22, 36). Our analysis, incorporating data from five observational studies involving over 560,000 women, showed a significant 33% reduction in VTE risk among users of E2-based COCs compared to those using EE-based COCs (**Figure 2**).

The association of EE with LNG has been regarded as the safest option for COCs for over two decades at least, underpinned by a comprehensive foundation of clinical experience, epidemiological evidence, and pharmacological understanding (2). The preference for this combination stems from several critical factors that underscore its safety and efficacy (14). EE/LNG combination has been consistently associated with a lower risk of VTE compared to COCs that include newer progestogens such as desogestrel (DSG), gestodene (GSD), or DRSP. This assertion is supported by numerous epidemiological studies and systematic reviews, highlighting a reduced relative risk of VTE, which has been a pivotal factor in its widespread acceptance and use (6, 7, 37–40). The safety profile of levonorgestrel, as one of the earliest progestogens used in COCs, is therefore well-documented and extensively studied, offering a rich data set on its long-term safety, efficacy, and tolerability. This extensive history of usage has facilitated a deep understanding of the potential risks and benefits associated with its use, contributing to its reputation as a reliable contraceptive option.

Importantly, EE/LNG COCs have also shown to exert a lesser impact on coagulation factors and the hemostatic system compared to combinations containing EE in association with other progestogens (41, 42). This reduced influence on coagulation pathways plays a significant role in the lower observed risk of thrombotic events, further solidifying its status as a safer option in COC containing EE as estrogenic component (42). Additionally, public health agencies and regulatory bodies, including the EMA, have conducted thorough evaluations of the thrombotic risks associated with different types of COCs. These assessments have consistently identified the EE/LNG combination as having a favorable benefit-risk profile, particularly in relation to thrombotic risks (4, 8).

Although the lowest, the absolute risk of VTE with EE/LNG remains associated with >2-fold increased risk compared to non-use of COC (6). As nicely estimated by epidemiological studies, reducing from EE 50 mcg to 20–30 mcg permits to decrease the risk of VTE of LNG-containing COCs from >6-fold to ± 2-fold (6). Nevertheless and importantly, not only the dose of EE is important but also the dose and the type of the associated progestin drives the risk (5). Of note, when used at the dose of 50 mcg, EE was associated with LNG at the dose of 125 mcg while it was 100 mcg and 150 mcg for EE 20 mcg and 30 mcg, respectively. The association is therefore of importance when considering the overall impact on coagulation since it implies an equilibrium between the pharmacodynamic properties of both the estrogen and the progestin present in the preparation (2). Such mechanistical explanation also permits to understand why some progestin like dienogest expresses an important risk once associated with EE (43) while it is lower with E2 (11).

Previous biological data suggested a similar to lower risk of VTE associated with natural estrogens like E2 or E4. Extensive evaluations of the coagulation cascade have been undertaken with these natural estrogens compared to the synthetic alternative. Most biomarkers which were sensitive toward changes induced by CHCs revealed the natural estrogens were less prone to induce changes compared to ethinylestradiol containing pills, including the reference EE/LNG (2, 17, 18, 20, 22, 36). More specifically, a specific biomarker which is able to capture and integrate most of the coagulation changes induced by estrogenic components (44) shows a distinctive effect of natural estrogens compared to EE containing COCs (45). Namely, the normalized activated protein C sensitivity ratio (nAPCsr) was able to demonstrate that EE-based COCs exhibit higher APC resistance compared to natural estrogens like E2 or E4 (46). Our group and others previously suggest that E2 and E4 compounds may offer a similar to even lower risk of cardiovascular events, including VTE, when compared to EE. This evidence aligns with the findings of this meta-analysis and supports the reduced VTE risk associated with natural estrogen based COCs.

Our meta-analysis suggests that contraceptives based on E2 may be associated with a reduced risk of VTE compared to those containing EE/LNG. The unadjusted OR of 0.80 does not reach statistical significance, indicating only a modest trend toward risk reduction. However, this trend becomes statistically significant in the context of adjusted HRs, which range from 0.49 to 0.68 across various sensitivity analyses. This significant reduction is exemplified by data from two methodologically similar ZEG studies, which demonstrate a 49% decrease in VTE risk (adjusted HR 0.51, 95% CI 0.29–0.90). These studies collectively represent over 150,000 women-years of data (13, 23).

Crucially, these findings highlight the substantial impact of confounding factors on VTE risk assessments in populations using natural estrogen-based contraceptives. The consistently lower adjusted HRs compared to their corresponding crude ORs suggest that any observed non-significant reduction with crude ORs must be interpreted with caution. These unadjusted measures likely conceal the true extent of the protective effects offered by natural estrogens compared to EE/LNG, due to insufficient adjustment for known confounding variables. This analysis underscores the importance of considering adjusted measures when evaluating the VTE risk associated with different hormonal contraceptives.

The studies included in this meta-analysis exhibit significant differences in design, sample size, population characteristics, and outcomes. Lidegaard (37) and Bauerfeind (23) are large prospective studies with sample sizes exceeding 70,000 women, while Schink (25) and Heikinheimo (24) are smaller case-control studies, providing more detailed demographic insights. Among all the studies, only the study of Lidegaard shows an opposite trend. This study has not been published and peer-reviewed yet, and no information was available on the included population and the adjustment of potential confounders. The NOS scale was also not possible to determine. Beside this study, the trend observed in all other studies was similar, supporting our findings. Funding sources range from pharmaceutical companies (Reed, Bauerfeind (13, 23)), private foundation (Heikinheimo (24)) to governmental bodies (Schink (25)), influencing the study’s perceived biases. Quality assessments using the NOS scale show high ratings (9/9 stars) for Reed (13), Schink (25), and Bauerfeind (23), indicating robust methodologies.

As a limited number of studies met our inclusion criteria, we decided to investigate whether our data fits with the effect size observed with well-documented previous investigations. In the 3 studies included in our meta-analytical model having compared different EE preparations, we investigate the OR of oCOC versus EE-LNG. We observed a trend toward a lower risk with EE/LNG which is not statistically significant probably due to the lower size of this subcomparison. However, the ±30% increased risk observed with oCOC is in line with the literature reporting comparison between EE/LNG and oCOC (47). Oedingen et al. reported a risk ratio comprised between 1.18 (EE20/GSD) and 1.46 (EE30–40/DSG) in their systematic review and meta-analysis aiming to compare EE/LNG versus oCOC (47). This comforts our observation that, despite the low number of studies included in our meta-analysis, the estimates are in line with previous studies strengthening our observation that E2-based COC demonstrated lower risk of VTE than EE-based COCs.

One limitation of this study is the generalization to other natural estrogens since we only were able to retrieve studies comparing E2 with EE-based pill. No direct comparisons are available to date with E4/DRSP. However, even if no direct comparison between E4 and EE has been made in a large observational study, the incidence of VTE observed with E4/DRSP during the phase-3 clinical program was 3.7/10,000 women-year, largely below the ones reported these last years in PASS evaluating EE-based COCs (**Table 3**). Other recent randomized trials were in line with these higher incidences for EE containing pills, transdermal patch, or vaginal ring (48–50). Another limitation resides in the fact that only a limited number of studies met our inclusion criteria. Nevertheless, the five studies included a total of over 500,000 patients, and their results consistently supported our conclusion. Additionally, fewer patients were included in the E2 arms compared to the EE arms, reflecting the real-life usage of COC. Nonetheless, further research is needed to strengthen our findings.

It thus becomes more and more evident that the choice of estrogen in COCs is a critical determinant of cardiovascular safety, challenging current prescribing practices and inviting a re-evaluation of guidelines to prioritize patient safety. The potential for natural estrogens like E2 and E4 to offer safer alternatives, without compromising contraceptive effectiveness, necessitates a paradigm shift in contraceptive prescribing practices.

## Conclusions

In conclusion, the evidence from our systematic review, supported by several previous and independent evaluation of the haemostatic impact of COCs, advocates for a re-evaluation of first-line therapy in contraception toward safer alternatives, prioritizing the use of natural estrogens. This shift not only addresses the immediate concern of reducing VTE risk but also aligns with a broader commitment to enhancing women’s health outcomes by ensuring access to safe, effective contraceptive options. As we move forward, integrating these insights into clinical practice will be crucial, guiding the selection of COCs based on a holistic understanding of their safety profile, particularly regarding thrombotic risk.

## Data Availability

The datasets presented in this study can be found in online repositories. The names of the repository/repositories and accession number(s) can be found below https://osf.io/n9dav/.
