# Supplementary material for: Are natural estrogens used in contraception at lower risk of venous thromboembolism than synthetic ones? A systematic literature review and meta-analysis
Source: Front Endocrinol (Lausanne). 2024 Aug 16;15:1428597. doi: 10.3389/fendo.2024.1428597 (PMC11362054; doi:10.3389/fendo.2024.1428597)
Supplement: Supplementary file 1 [file DataSheet1.docx]

**Appendices**

All data are available at <https://osf.io/n9dav/> and the full research strategy is presented below.

**A1. Search strategies for Medline (Ovid) and Embase databases**

**Database: Ovid MEDLINE(R) ALL <1946 to November 29, 2023>**
1  Estradiol/ (86384)
2  estradiol*.ti,ab,kf. (90617)
3  oestradiol*.ti,ab,kf. (13849)
4  Estetrol/ (145)
5  estetrol*.ti,ab,kf. (214)
6  oestetrol*.ti,ab,kf. (12)
7  Ethinyl Estradiol/ (9041)
8  (ethinylestradiol* or "ethinyl estradiol*").ti,ab,kf. (7735)
9  (ethinyloestradiol* or "ethinyl oestradiol*").ti,ab,kf. (816)
10  Estrogens/ (61855)
11  estrogen*.ti,ab,kf. (162540)
12  oestrogen*.ti,ab,kf. (23402)
13  or/1-12 (272433)
14  Contraception/ (22365)
15  contracept*.ti,ab,kf. (81733)
16  Contraceptive Agents/ (6266)
17  Contraceptive Devices/ (1554)
18  Contraceptives, Oral/ (19711)
19  (birth adj3 pill*).ti,ab,kf. (642)
20  or/14-19 (92527)
21  exp Thrombosis/ (152161)
22  thromb*.ti,ab,kf. (509586)
23  (blood adj1 clot*).ti,ab,kf. (12054)
24  VTE.ti,ab,kf. (15795)
25  DVT.ti,ab,kf. (13342)
26  thromboemboli*.ti,ab,kf. (79342)
27  Thromboembolism/ (25682)
28  Venous Thrombosis/ or Venous Thromboembolism/ (44074)
29  phlebothrombos*.ti,ab,kf. (561)
30  Cardiovascular System/ (36417)
31  ((cardiovascular or circulatory) adj2 system*).ti,ab,kf. (43478)
32  or/21-31 (636976)
33  Case-Control Studies/ (330823)
34  Epidemiologic Studies/ (9441)
35  Cohort Studies/ (335070)
36  Clinical Trial/ (539095)
37  ((case-control or cohort or epidemiolog* or clinical or observationnal) adj1 stud*).ti,ab,kf. (760756)
38  or/33-37 (1696311)
39  13 and 20 and 32 and 38 (387)
40  Menopause/ (30302)
41  Postmenopause/ (27940)
42  menopaus*.ti,ab,kf. (58429)
43  postmenopaus*.ti,ab,kf. (63028)
44  or/40-43 (115447)
45  39 not 44 (339)
46  45 not (exp animals/ not humans.sh.) (337)
47  limit 46 to yr="2000 -Current" (139)

**Database: Embase**


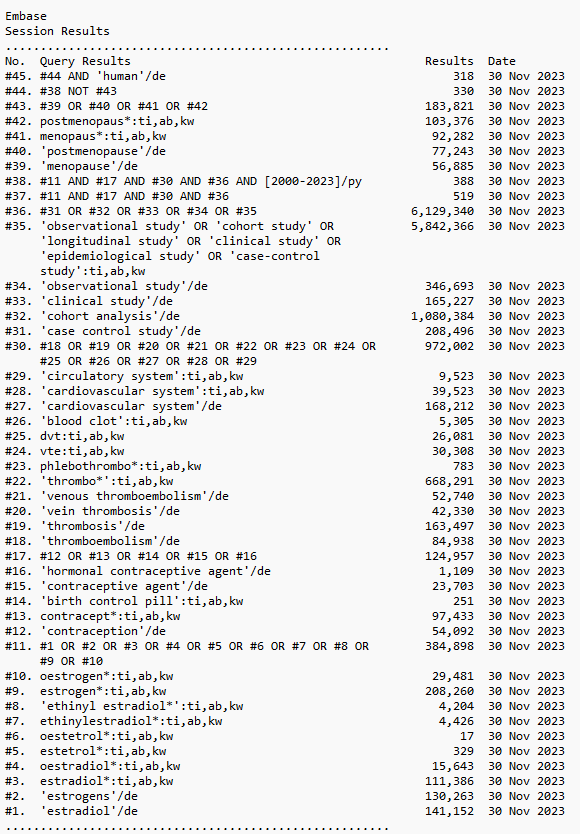


**A2. Additional Figures**

**
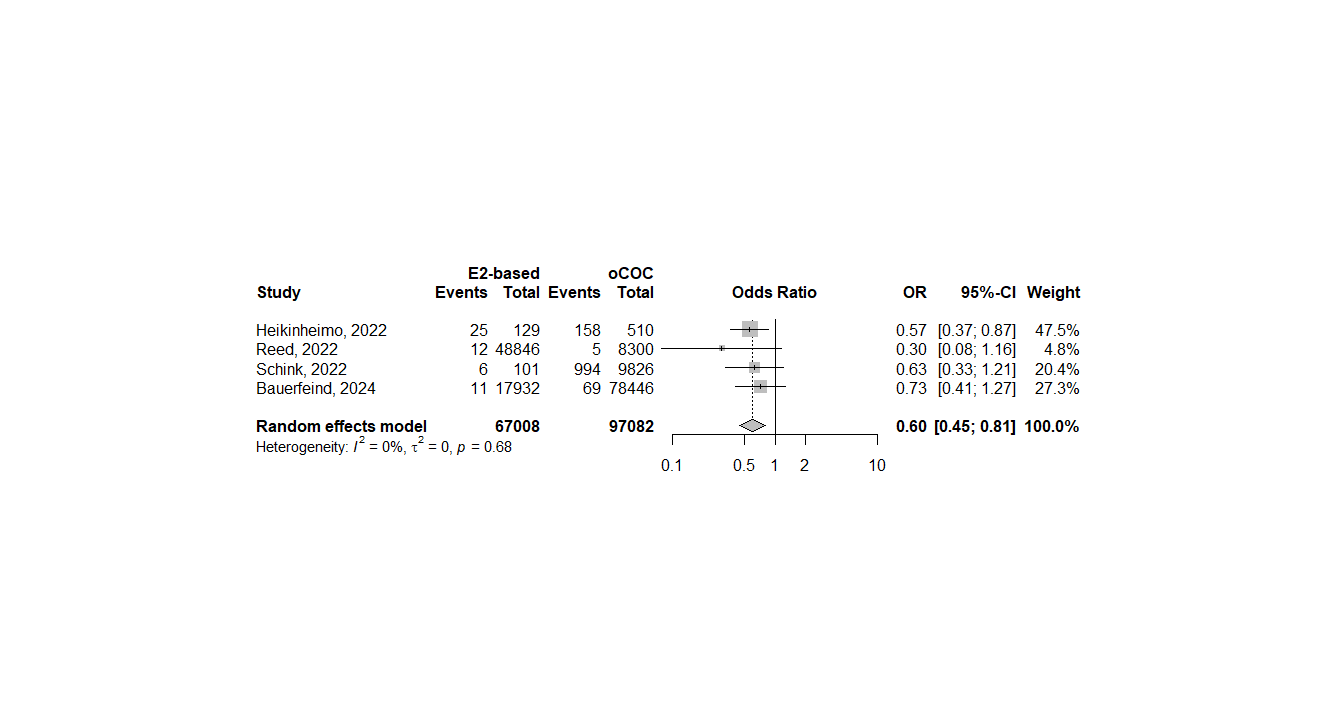
**

**Figure A1.** E2-based vs oCOC

**
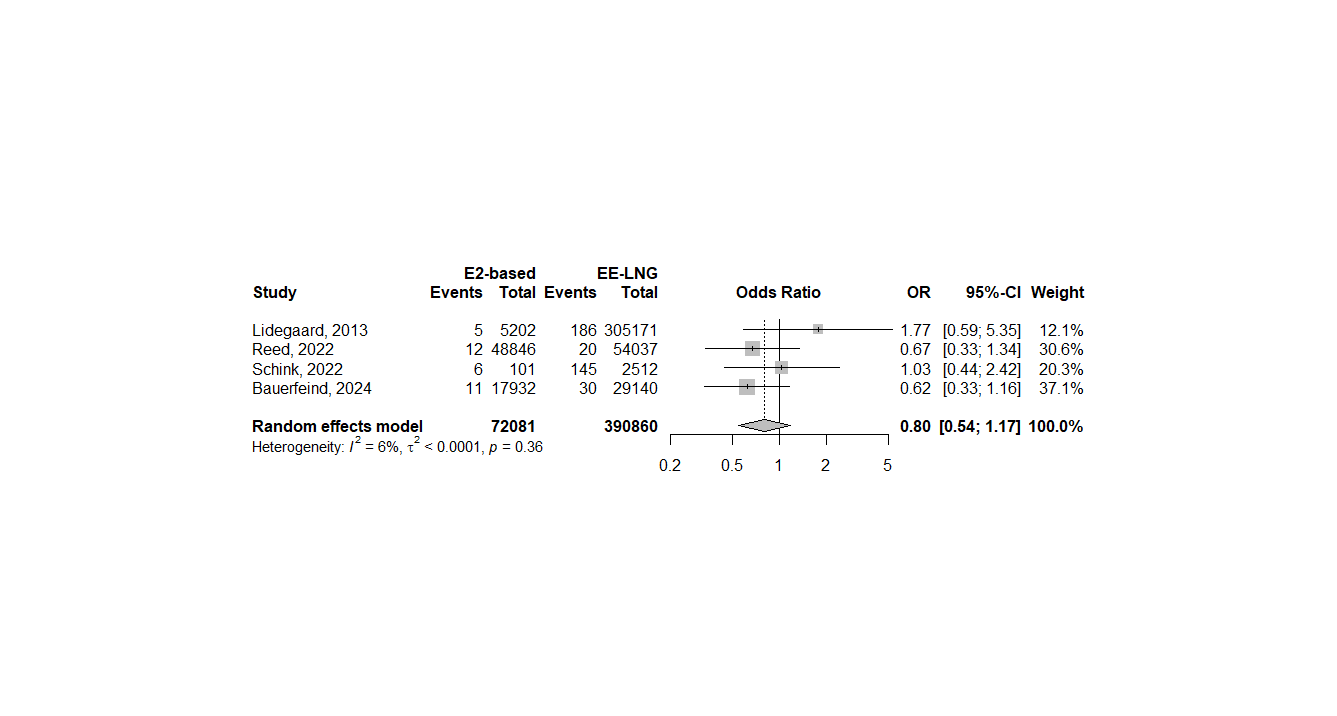
**

**Figure A2**. E2-based vs EE/LNG

**
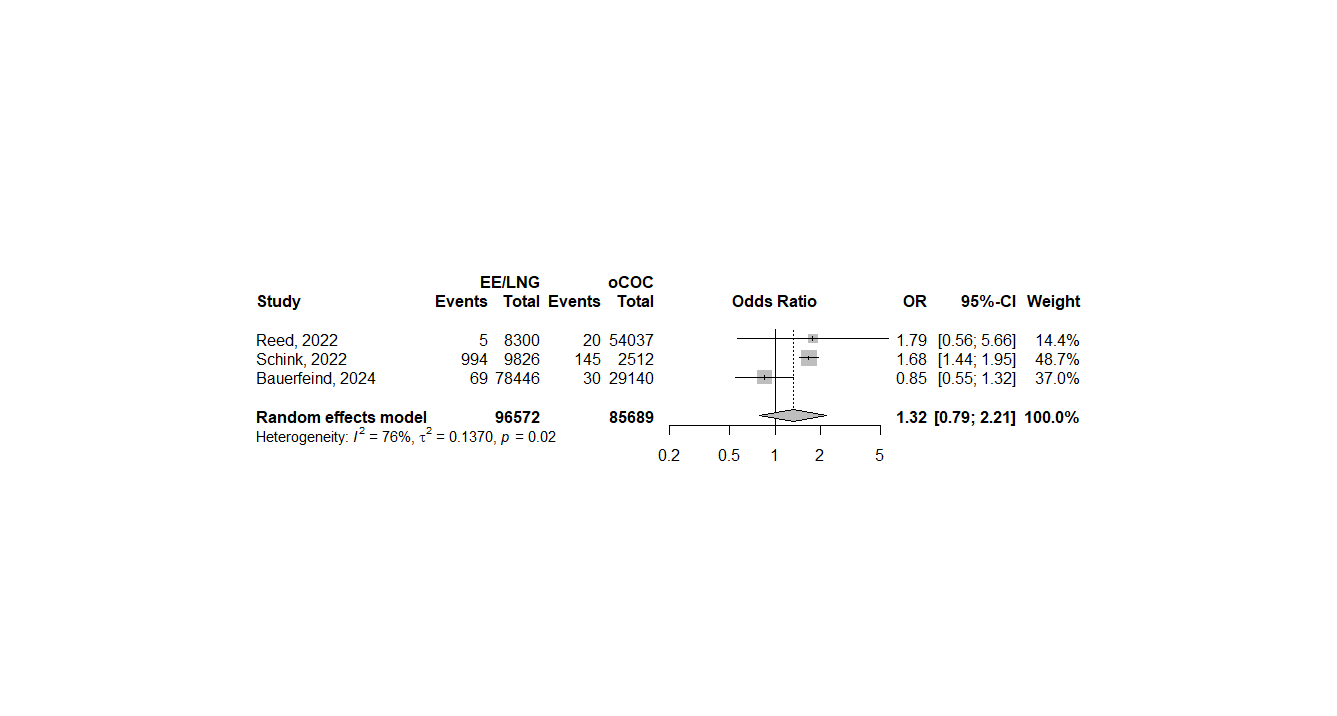
**

**Figure A3**. EE/LNG vs oCOC


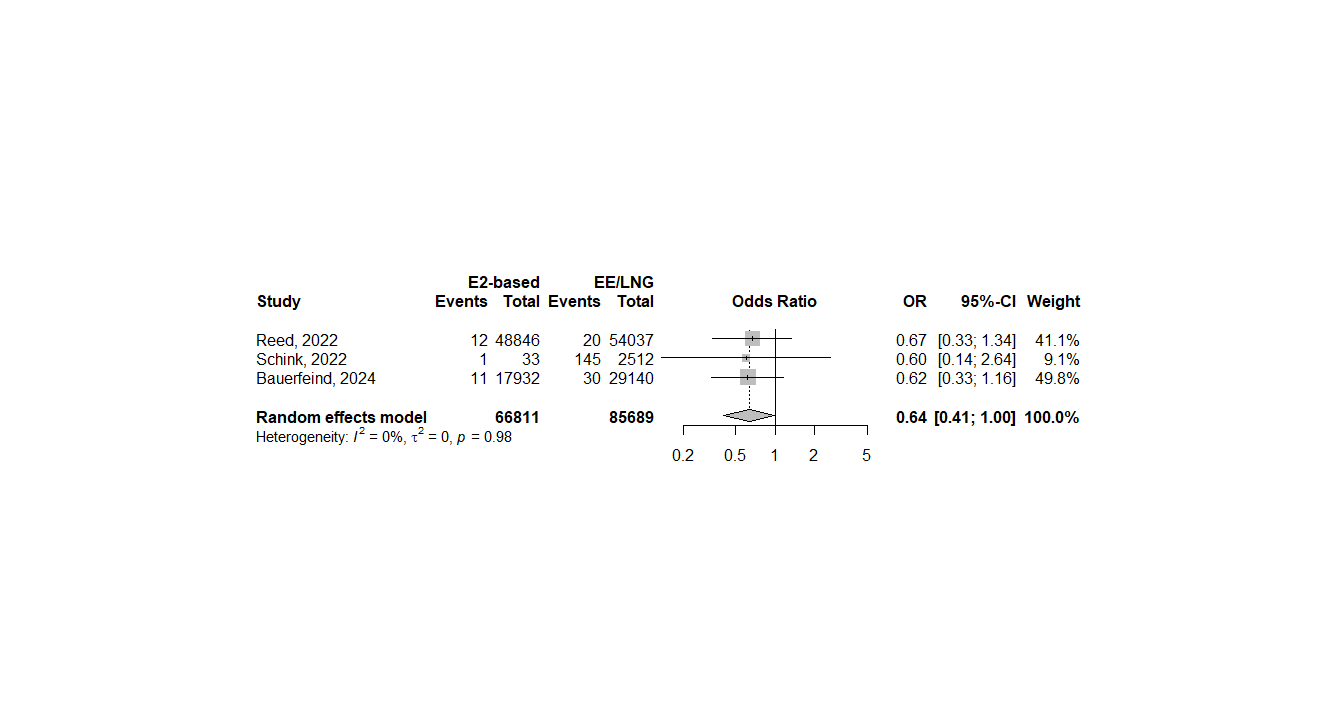


**Figure A4**. E2-based vs EE/LNG in studies with adjustment for cofounding factors E2/DNG - crude analyses

**
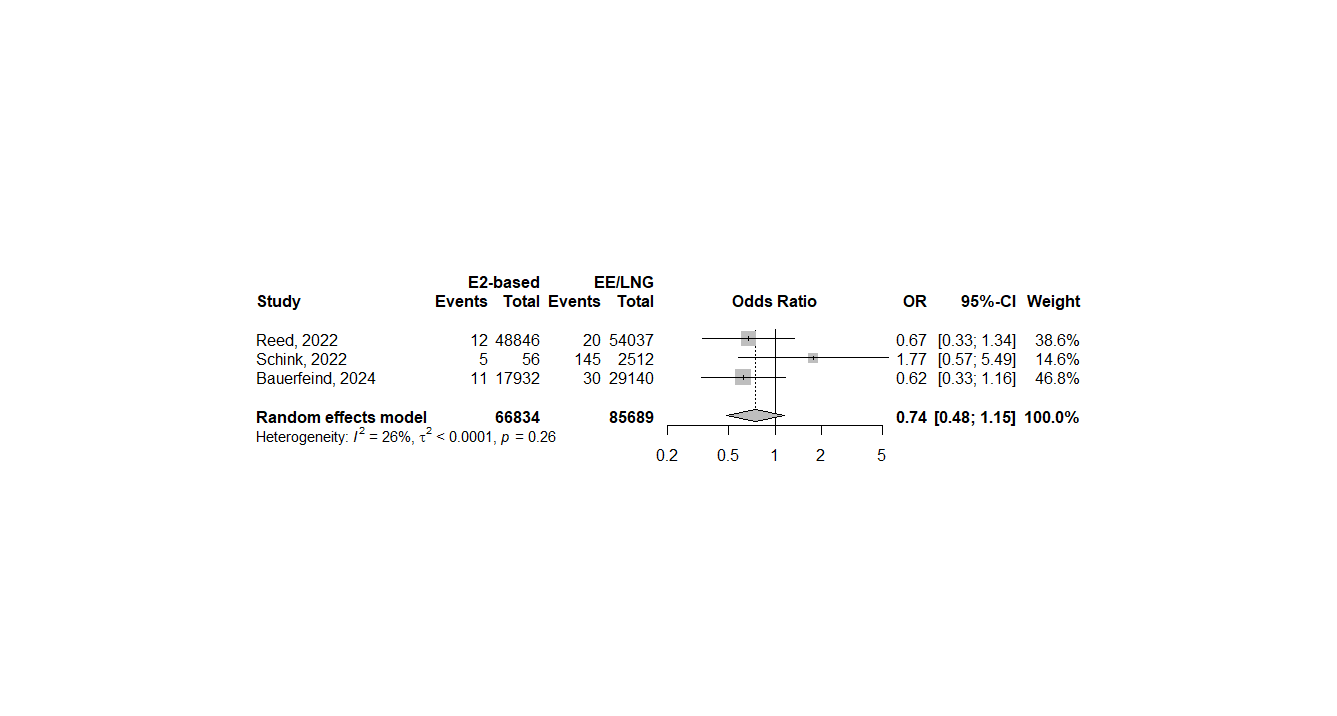
**

**Figure A5**. E2-based vs EE/LNG in studies with adjustment for cofounding factors E2/NOMAC - crude analyses


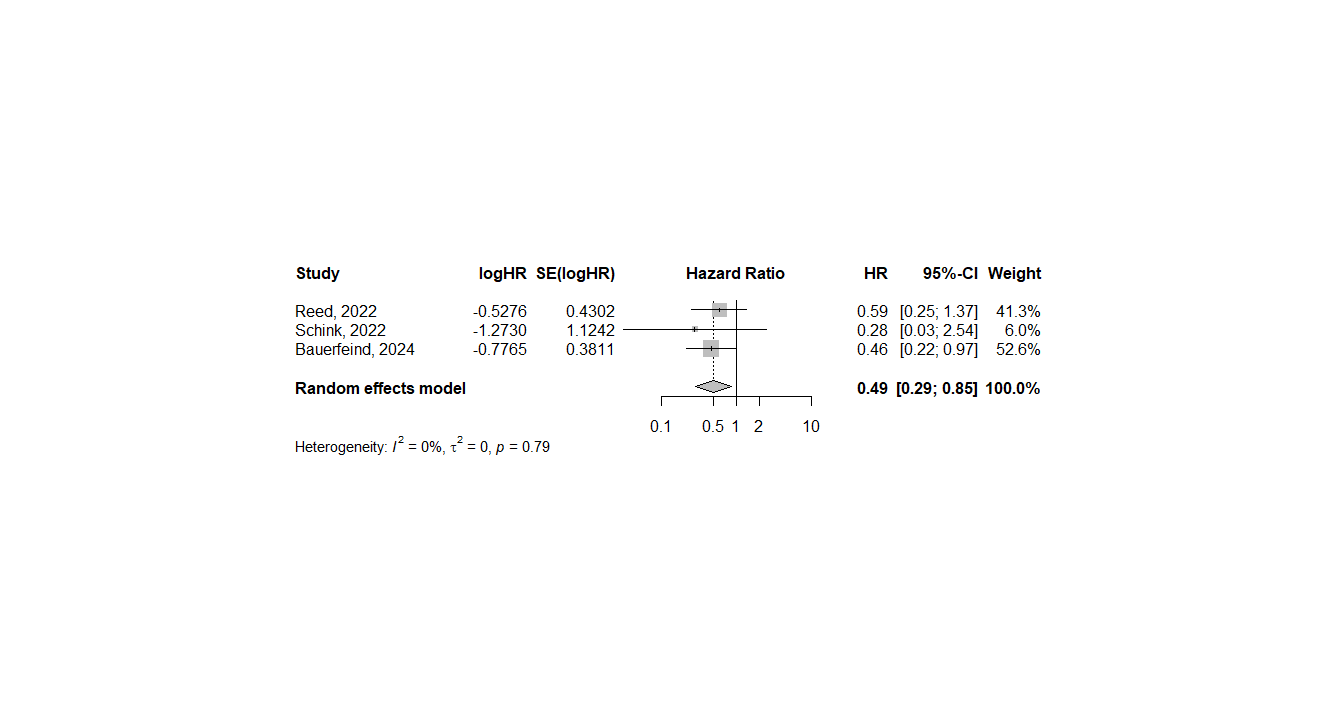


**Figure A6.** E2-based vs EE/LNG in studies with adjustment for cofounding factors E2/DNG - adjusted analyses


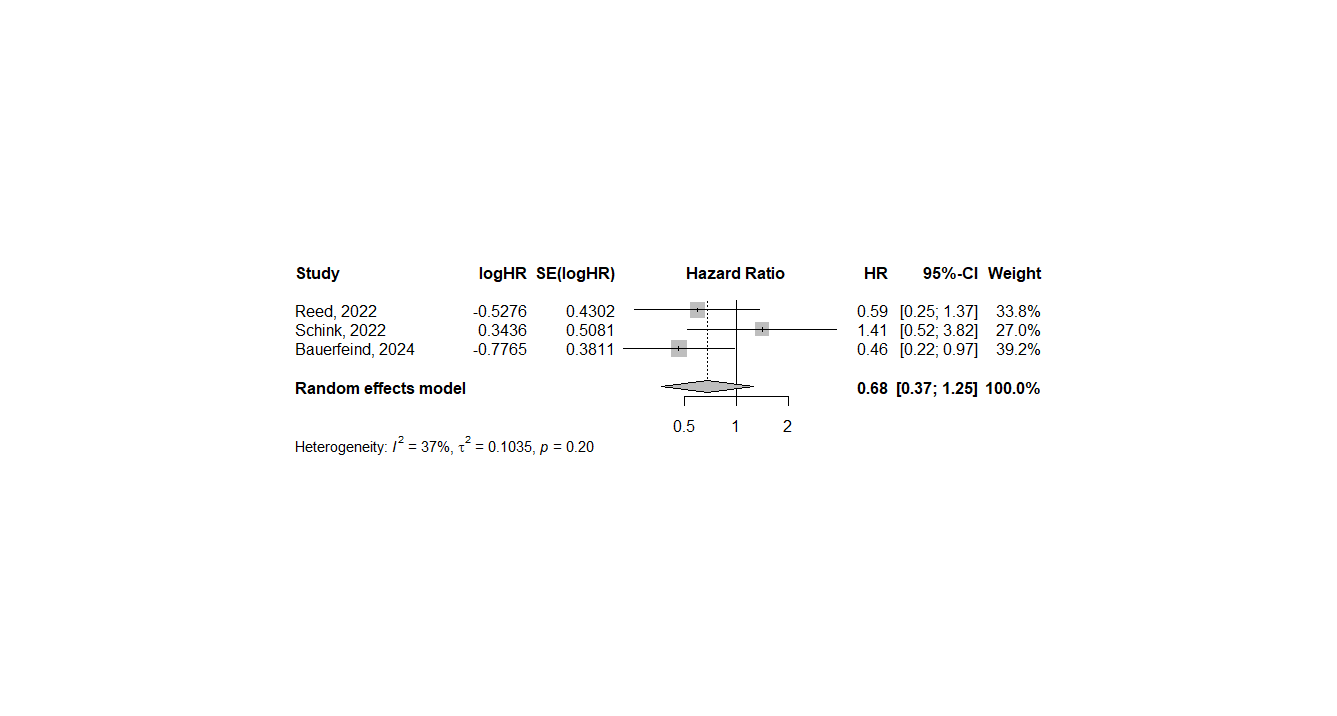


**Figure A7.** E2-based vs EE/LNG in studies with adjustment for cofounding factors E2/NOMAC – adjusted analyses


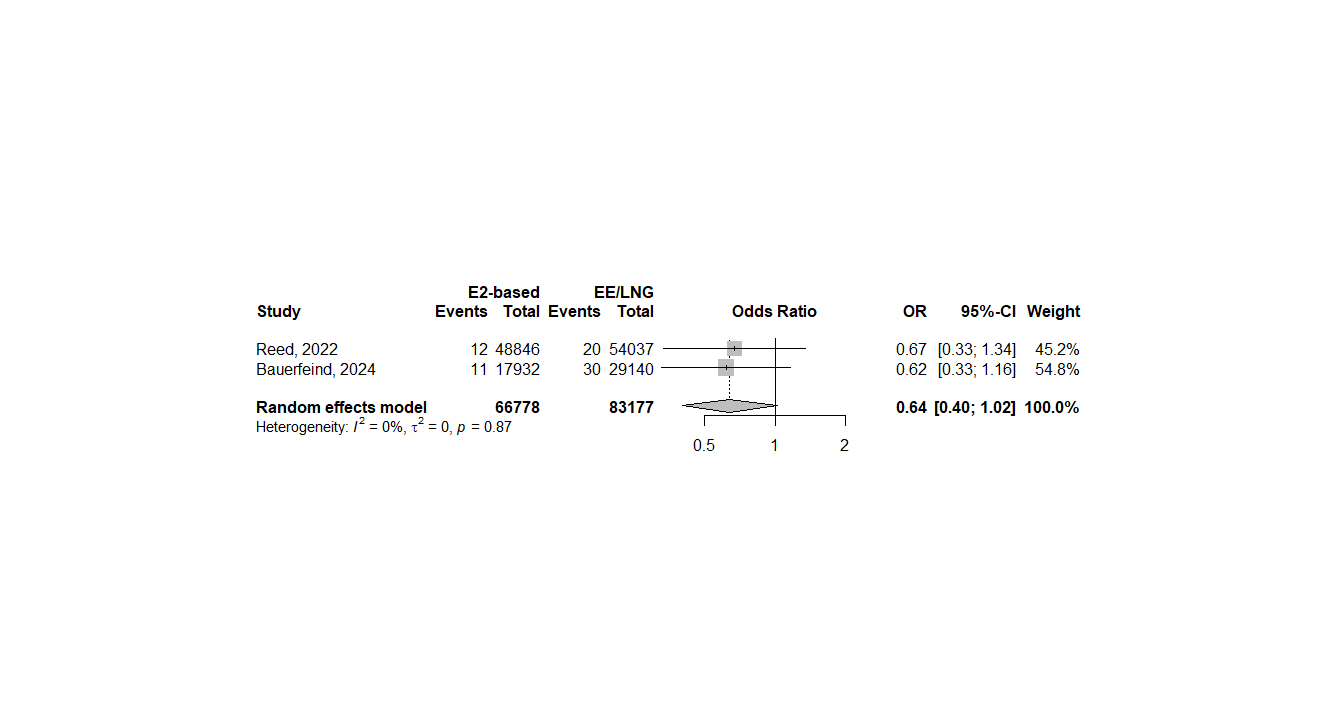


**Figure A8**. E2-based vs EE/LNG (studies from ZEG) – crude analyses


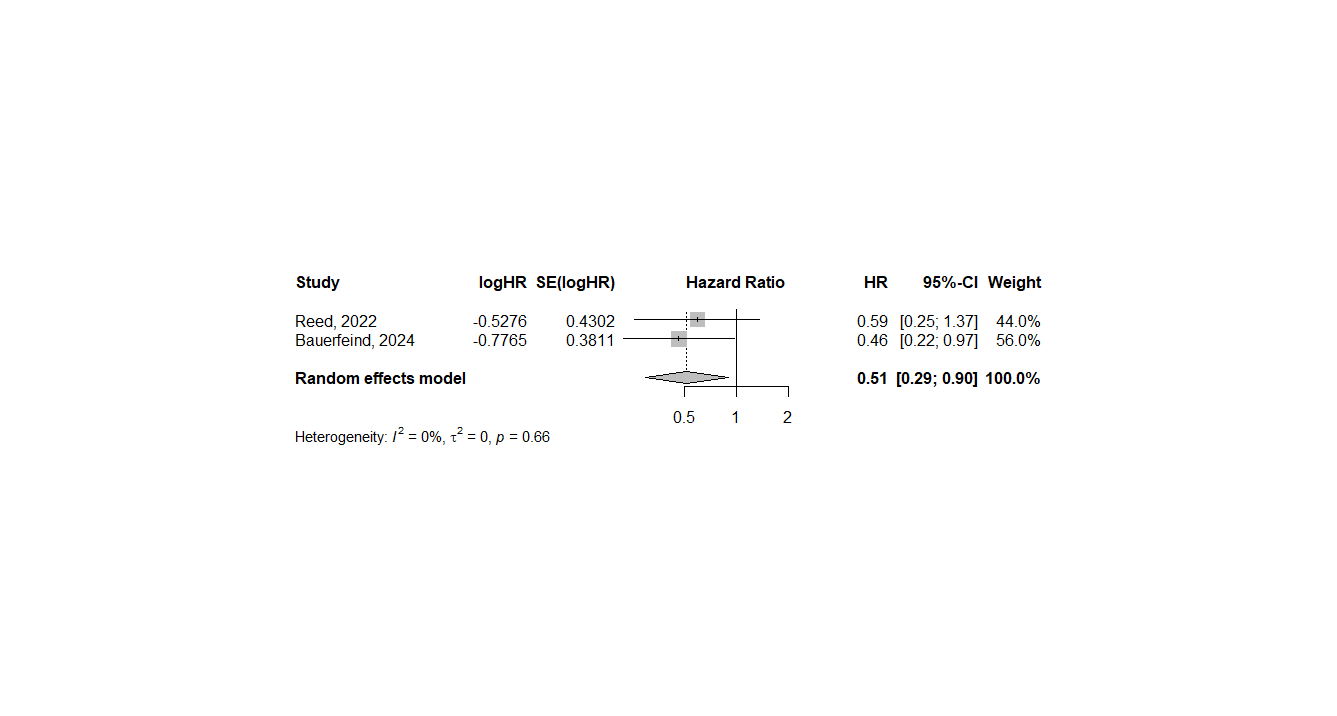


**Figure A9.** E2-based vs EE/LNG (studies from ZEG) – adjusted analyses
